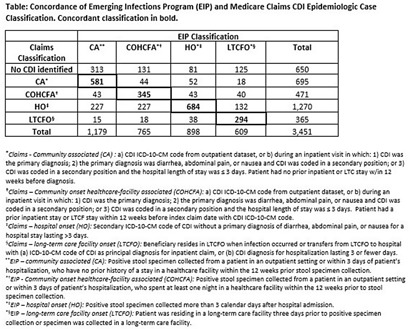# Comparison of Medicare Claims-based Clostridioides difficile infection classification to chart review using a linked cohort

**DOI:** 10.1017/ash.2024.180

**Published:** 2024-09-16

**Authors:** Dustin Currie, Chantal Lewis, Kelly Hatfield, Joseph Lutgring, Sophia Kazakova, James Baggs, Lauren Korhonen, Maria Correa, Dana Goodenough, Danyel Olson, Jill Szydlowski, Jasmine Watkins, Ghinwa Dumyati, Scott Fridkin, Christopher Wilson, Sujan Reddy, Alice Guh

**Affiliations:** Centers for Disease Control and Prevention; Connecticut Emerging Infections Program; Georgia Emerging Infections Program/Foundation for Atlanta Veterans Education and Research/Atlanta VA Medical Center; Connecticut Emerging Infections Program, Yale School of Public Health; University of Rochester Medical Center; Tennessee Department of Health; Emory Healthcare and Emory University; Tennessee Department of Health

## Abstract

**Background:** Medicare claims are frequently used to study Clostridioides difficile infection (CDI) epidemiology. Categorizing CDI based on location of onset and potential exposure is critical in understanding transmission patterns and prevention strategies. While claims data are well-suited for identifying prior healthcare utilization exposures, they lack specimen collection and diagnosis dates to assign likely location of onset. Algorithms to classify CDI onset and healthcare association using claims data have been published, but the degree of misclassification is unknown. **Methods:** We linked patients with laboratory-confirmed CDI reported to four Emerging Infections Program (EIP) sites from 2016-2020 to Medicare beneficiaries using residence, birth date, sex, and hospitalization and/or healthcare exposure dates. Uniquely linked patients with fee-for-service Medicare A/B coverage and complete EIP case report forms were included. Patients with a claims CDI diagnosis code within ±28 days of a positive CDI test reported to EIP were categorized as hospital-onset (HO), long-term care facility onset (LTCFO), or community-onset (CO, either healthcare facility-associated [COHCFA] or community-associated [CA]) using a previously published algorithm based on claim type, ICD-10-CM code position, and duration of hospitalization (if applicable). EIP classifies CDI into these categories using positive specimen collection date and other information from chart review (e.g. admit/discharge dates). We assessed concordance of EIP and claims case classifications using Cohen’s kappa. **Results:** Of 10,002 eligible EIP-identified CDI cases, 7,064 were linked to a unique beneficiary; 3,451 met Medicare A/B fee-for-service coverage inclusion criteria. Of these, 650 (19%) did not have a claims diagnosis code ±28 days of the EIP specimen collection date (Table); 48% (313/650) of those without a claims diagnosis code were categorized by EIP as CA CDI. Among those with a CDI diagnosis code, concurrence of claims-based and EIP CDI classification was 68% (κ=0.56). Concurrence was highest for HO and lowest for COHCFA CDI. A substantial number of EIP-classified CO CDIs (30%, Figure) were misclassified as HO using the claims-based algorithm; half of these had a primary ICD-10 diagnosis code of sepsis (226/454; 50%). **Conclusions:** Evidence of CDI in claims data was found for 81% of EIP-reported CDI cases. Medicare classification algorithms concurred with the EIP classification in 68% of cases. Discordance was most common for community-onset CDI patients, many of whom were hospitalized with a primary diagnosis of sepsis. Misclassification of CO-CDI as HO may bias findings of claims-based CDI studies.

**Figure:**
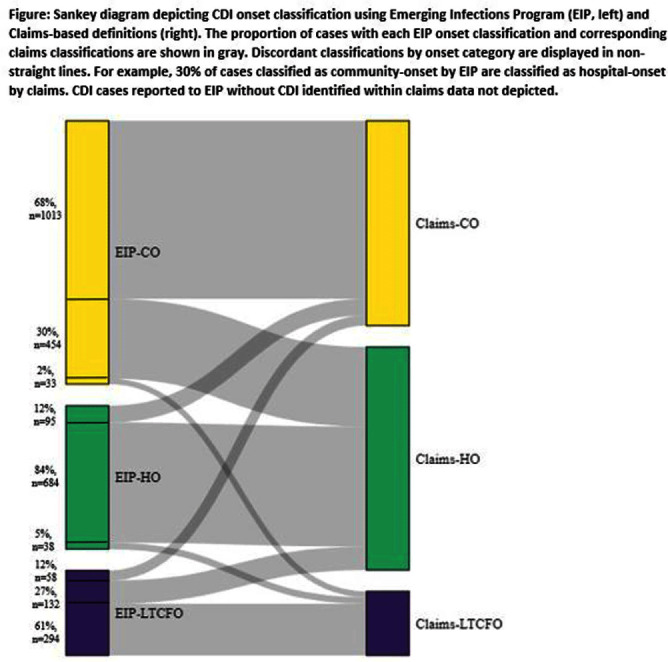

**Figure:**